# User Experience of 7 Mobile Electroencephalography Devices: Comparative Study

**DOI:** 10.2196/14474

**Published:** 2019-09-03

**Authors:** Thea Radüntz, Beate Meffert

**Affiliations:** 1 Mental Health and Cognitive Capacity Federal Institute for Occupational Safety and Health Berlin Germany; 2 Department of Computer Science Humboldt-Universität zu Berlin Berlin Germany

**Keywords:** wearable devices, user experience, electroencephalography, mobile applications, electrodes, dry electrodes

## Abstract

**Background:**

Registration of brain activity has become increasingly popular and offers a way to identify the mental state of the user, prevent inappropriate workload, and control other devices by means of brain-computer interfaces. However, electroencephalography (EEG) is often related to user acceptance issues regarding the measuring technique. Meanwhile, emerging mobile EEG technology offers the possibility of gel-free signal acquisition and wireless signal transmission. Nonetheless, user experience research about the new devices is lacking.

**Objective:**

This study aimed to evaluate user experience aspects of emerging mobile EEG devices and, in particular, to investigate wearing comfort and issues related to emotional design.

**Methods:**

We considered 7 mobile EEG devices and compared them for their wearing comfort, type of electrodes, visual appearance, and subjects’ preference for daily use. A total of 24 subjects participated in our study and tested every device independently of the others. The devices were selected in a randomized order and worn on consecutive day sessions of 60-min duration. At the end of each session, subjects rated the devices by means of questionnaires.

**Results:**

Results indicated a highly significant change in maximal possible wearing duration among the EEG devices (χ^2^_6_=40.2, n=24; *P*<.001). Regarding the visual perception of devices’ headset design, results indicated a significant change in the subjects’ ratings (χ^2^_6_=78.7, n=24; *P*<.001). Results of the subjects’ ratings regarding the practicability of the devices indicated highly significant differences among the EEG devices (χ^2^_6_=83.2, n=24; *P*<.001). Ranking order and posthoc tests offered more insight and indicated that pin electrodes had the lowest wearing comfort, in particular, when coupled with a rigid, heavy headset. Finally, multiple linear regression for each device separately revealed that users were not willing to accept less comfort for a more attractive headset design.

**Conclusions:**

The study offers a differentiated look at emerging mobile and gel-free EEG technology and the relation between user experience aspects and device preference. Our research could be seen as a precondition for the development of usable applications with wearables and contributes to consumer health informatics and health-enabling technologies. Furthermore, our results provided guidance for the technological development direction of new EEG devices related to the aspects of emotional design.

## Introduction

### User Experience Research of Emerging Electroencephalography Technology

In the previous years, registration of brain activity has become more and more popular not only in science but also in the home and gaming sector. Users look forward to identifying and quantifying their mental state directly there where human information processing takes place, and electroencephalography (EEG) offers a way to assess the levels of fatigue, stress, or emotions. The state feedback can then be used to prevent undesired situations, enhance wanted effects, or control devices. The increasing number of publications related to brain-computer interfaces [1-7] indicates an ever-growing interest in communication systems where encoded brain activity from the user is used as an alternative channel to send information to a computer. In addition, progress in sensor technology enables the production of low-cost, light-weighted, and marketable devices. However, extended use of the EEG is hampered by user experience challenges and user acceptance issues regarding the measuring technique.

Only a few years ago, one of the main issues was the limited mobility of the subjects because of the wired connections going from the electrode cap to an amplifier and computer. Meanwhile, wireless signal transmission helps to overcome this problem and allows subjects to move more freely. Further concerns are related to the application of gel electrodes and skin preparation for reducing the impedance. Emerging sensor technology uses gel-free sensors to enable a quick and easy application of the electrodes by the users themselves. For assuring an acceptable signal quality, impedance between electrodes and skin must be low, that is, electrodes need a good and permanent contact to the skin. This becomes particularly difficult to achieve for dry electrodes that work without the conductive gel. Given this, the question of wearing comfort and user experience becomes even more evident.

Finally, there are also user experience issues related to the unflattering visual appearance of the traditional EEG caps and thus linked to the research field of emotional design [8]. The core idea thereby is that products’ design strives to elicit positive emotions and thus influence users’ perception to provide a greater level of user experience. The 3-level model of emotional design includes the visceral, the behavioral, and the reflective level [8,9]. The visceral is the most basic, immediate level and addresses our first reactions to visual or sensory aspects (eg, aesthetics and quality) of the product. The behavioral level refers to usability aspects of the product, whereas the reflective level comprises conscious cognition. More general, the reflective level asks how well the product fits in with user’s current self-image and addresses not only mental and emotional but also social aspects.

To recap, there is growing interest among users in brain state monitoring and increased efforts by developers for developing mobile EEG devices. However, serious user experience research in this field is rare, and it remains still unclear whether user acceptance of the new devices is improved compared with traditional EEG technology. In our study, we aimed to address this issue and advance the state of the art regarding user experience of emerging EEG devices. Thereby, we focused on the wearing comfort of the devices and aspects of emotional design, particularly the behavioral and reflective levels.

### Related Work

During the previous years, the advances in sensor technology promoted the research regarding the usability of emerging EEG devices. Most of the published papers concentrated only on device functionality and signal quality comparison between the traditional gel-based electrodes and the new dry electrodes [7,10-12].

Only a small number of studies were concerned with devices’ wearing comfort and design requirements. Nikulin et al [13] reported that for designing a new kind of electrodes, they considered not only signal quality but also electrodes’ visual appearance and wearing comfort. They put effort to create extremely light and small electrodes that could be applied with some conductive gel directly on the head without any cap or headset. During the study, subjects reported that the electrodes were not noticeable and also not visually detectable by other people. Subjects felt less watched and thus better. Nikulin et al argued that this was particularly important when working outside the laboratory, and subjects were asked to behave naturally and free, in particular, during field experiments in real work environments. However, the main limitation was that the electrodes had to be applied with gel. This application procedure was time consuming and required specific knowledge about electrodes’ precise positions on the head. Hence, it had to be done by an experienced investigator and could not be done by the subject itself. A further limitation was that the subjects did not have the opportunity to compare the new electrode device with another.

Similarly, Grozea et al [14] reported on their work on new electrodes with fine, flexible, and metal-coated polymer bristles. The bristles should allow for a good contact through the hair, and simultaneously, they should be comfortable during wearing. The researchers tested the electrodes on subjects (ie, colleagues) that had previous experience with other kinds of electrodes (eg, gel-based and pin electrodes). The subjects concluded that although the bristles electrodes were better than the pin electrodes, the bristles could have been softer and more flexible to increase comfort. Limitations of the study were the small number of subjects participating and the lack of direct comparison among the different kinds of electrodes instead of recalling the wearing comfort from previous experiences.

Comparison studies among different commercial EEG devices regarding user experience were rare. A study by Ekandem et al [15] dealt with the comparison between Emotiv’s EPOC device and NeuroSky’s MindWave device. Research questions concerned the wearing comfort, the preparation, and the application time. The latter was less than 5 min for both devices and thus clearly less compared with traditional EEG devices. After 15 min of wearing, subjects were asked to answer questions about the overall comfort of the worn device, the length of time they would be able to wear it, and the type of discomfort [15]. Thereby, the EPOC device was rated more comfortable compared with the MindWave device. A main limitation of the study concerned the wearing time of 15 min because this could be insufficient for determining discomfort issues.

A study by Izdebski et al [16] was divided into 2 similar experiments that tested in total 7 devices. Of 7 devices, 4 devices (g.tec’s g.SAHARA, Emotiv’s EPOC, ANT Neuro’s asalab, and Brain Products’ [Brain Products GmbH] actiCAP) were tested by 4 subjects, and the remaining 3 devices (BioSemi’s ActiveTwo, Cognionics’ Dry System, and Cognionics’ Wet System) were tested by 9 subjects. Duration of the sessions varied between 1 and 3 hours, and the usability was assessed at the end of each session by a questionnaire. Surveyed usability aspects were comfort, cap fit, mood, and movement restriction. Izdebski et al reported that the gel-based electrode headsets asalab and actiCAP induced general discomfort although participants did not report an unpleasant feeling under the cap nor a high pressure of the electrodes. Regarding cap fit, the ActiveTwo and systems without adjustment possibilities received negative ratings. The EPOC, g.SAHARA, and asalab devices yielded a more negative mood at the end of the session, whereas the wired systems asalab and actiCAP were rated as more movement restricting. A limitation of the study concerns the lack of a consistent within-subject design and the very different session durations.

Hairston et al [17] conducted a usability research experiment with a wearing time duration of 60 min. They compared 4 EEG devices: 3 wireless EEG systems (Emotiv’s EPOC, Advanced Brain Monitoring’s B-Alert X10, and QUASAR’s HMS) and 1 wired, laboratory-grade device (Bio-Semi’s ActiveTwo). The main user experience aspects they focused on, besides signal quality issues, were the adaptability of the devices to different head sizes, comfort, and subjects’ device preference. They found that subjects preferred the B-Alert X10 device more than the other 2 wireless systems although it had gel-based electrodes. Subjects reported that the gel-infused pads of the B-Alert X10 device were more comfortable than the others. Finally, Hairston et al stated that future work was needed to systematically study usability factors and improve development efforts of new systems.

To compare the usability of a brain-computer interface for communication, Nijboer et al [18] tested 3 different EEG headsets (g.tec’s g.SAHARA, Emotiv’s EPOC, and BioSemi’s ActiveTwo). Apart from signal quality, Nijboer et al also assessed the speed and ease of headset’s setup, subjects’ rating about their appearance with headset, comfort, and general device preference. Nijboer et al obtained the highest setup time for the gel-based ActiveTwo device, the best aesthetic ratings for the EPOC device, and the best comfort ratings for the gel-based ActiveTwo and pin-based g.SAHARA devices. Although the EPOC device yielded the worst ratings regarding comfort, it was the device of choice in the ranking of preference. Nijboer et al assumed that aesthetics and ease of use could be more important factors than comfort when it comes to preference ranking. They stated that more research was needed to understand which user experience aspects influence subjects’ preference choice.

Table 1 summarizes the above-mentioned studies in a symmetric presentation style. To conclude, considering that duration of registration sessions and thus device wearing can take a long time, comfort requirements are particularly important. Existing studies regarding the usability of EEG headsets indicated that for assuring user acceptance, devices should be lightweight, comfortable, not painful to wear, and with an unobtrusive design. However, limitations of these studies were a limited number of participants, lack of comparisons among different devices, or a too short wearing duration of the EEG headsets. Most of the studies focused primarily on wearing comfort and neglected user experience aspects such as emotional design. In our study, we considered these things and systematically compared 7 different EEG devices.

**Table 1:** Literature review regarding user experience of emerging electroencephalography technology.

Reference	Devices tested	Electrode type and number	Set size	Wearing duration	User aspects and items	Results
Nikulin et al 2010 [13]	Proprietary development, traditional EEG^a^ cap	Miniaturized C-electrodes with gel, 3; standard electrodes with gel, 3	4 subjects	40-60 min	Wearing comfort, tactile sensation, shame	No tactile sensations associated with C-electrode wearing, no negative emotional impact in the presence of others, and no discomfort
Grozea et al [14]	Proprietary development	Dry bristle electrodes; no information about number of electrodes	8 colleagues (2 of them excluded)	<1 hour	Comfort issues	Most subjects reported them to be more advanced than the previously known
Ekandem et al [15]	Emotiv’s EPOC, NeuroSky’s MindWave	Saline-based, 14; dry, 1	13 subjects (2 of them excluded)	15 min	Comfort and wearing duration	EPOC more comfortable; at least 20 min possible
Izdebski et al [16]	g.tec’s g.SAHARA, Emotiv’s EPOC, Cognionics’ Dry System, ANT Neuro’s asalab, Brain Products’ actiCAP, BioSemi’s ActiveTwo, and Cognionics’ Wet System	Dry, 32; saline-based, 14; dry, 64; gel, 128; gel, 64; gel, 128; gel, 64	4 subjects (g.SAHARA, EPOC asalab, and actiCAP); 9 subjects (ActiveTwo, Cognionics’ Dry System, and Cognionics’ Wet System)	4 subjects (2-3 hours); 9 subjects (1-2 hours)	Comfort, cap fit, mood, and movement restriction	asalab and actiCAP induced general discomfort although participants did not report unpleasant feeling under cap nor high pressure of electrodes; ActiveTwo and systems without adjustment possibilities received negative ratings regarding cap fit; EPOC, g.SAHARA, and asalab yielded a more negative mood at the end of the session; the wired systems asalab and actiCAP were rated as more movement restricting
Hairston et al [17]	Emotiv’s EPOC, Advanced Brain Monitoring’s B-Alert X10, QUASAR’s HMS, and BioSemi’s ActiveTwo	Saline-based, 14; gel, 9; dry, 9; gel, 64	16 subjects (3-4 of them excluded)	60 min	Comfort, preference	Most preferred: B-Alert; comfortable to wear
Nijboer et al [18]	g.tec’s g.SAHARA, Emotiv’s EPOC, BioSemi’s ActiveTwo	Dry, 8; saline-based, 14; gel, 32	13 subjects	~1 hour	Speed and ease of setup, appearance with headset, comfort, and general preference	Highest setup time for ActiveTwo; best aesthetic ratings for EPOC; best comfort ratings for ActiveTwo and g.SAHARA; in general, most preferred: EPOC

^a^EEG: electroencephalography.

### Research Objectives

As the registration of brain activity outside the laboratory becomes more popular, aspects of user experience attract more attention when new devices are to be developed. Apart from improving wearing comfort that is crucial regarding user experience, developers also put more emphasis on the headset design of the EEG devices. This can lead to extraordinary designs that are not always flattering and easy to use for the user. In such cases, the visual appearance and behavior of the device can influence the well-being of a person [13].

Our first research objective was concerned with the test of the devices. First, we referred to the well-known issue of wearing comfort linked to the different electrode types and the question of how comfortable the different electrodes were after a longer wearing time. We assumed that maximal possible wearing duration would vary significantly among the devices depending on the type of electrode. Spring-loaded or rigid pin electrodes were expected to apply more pressure on the head and thus to have a smaller comfort and a low possible wearing duration. Gel-based electrodes were expected to assure a better comfort and could be worn for longer. Furthermore, we were interested in testing the devices in regard to the visceral and behavioral levels of emotional design. These comprised the design of the devices and the ease of use. To this end, we formulated the following research questions for the evaluation of the devices:

Research question 1a: Does maximal possible wearing duration differ among devices with different electrode types?

Research question 1b: Does the visual perception of devices’ design differ among each other?

Research question 1c: Does practicability of the devices differ among each other?

Especially in cases where the EEG device is worn in public (eg, workplace), some users could prefer a more unobtrusive design. This can be linked to the reflective level of Norman’s 3-level model of emotional design [8]. Thereby, information from the visceral and behavioral levels are combined with our knowledge and experiences, filtered, and cognitively processed. At this level, user’s self-image plays a crucial role. Beyond the intended use of the product, user preferences are based on who will see it and how these viewers will judge the user with it.

Hence, we were interested to find out if users were willing to accept less comfort for a more attractive headset design. On the basis of this consideration, we formulated our second research objective:

Research question 2: Does visual appearance affect the overall rating of the devices more than wearing comfort?

In the Methods section of our study, we introduce the EEG devices, material used, sample set, and procedure for conducting the experiments. The gained results are presented in the Results section and discussed in the following section. Thereby, we mention potential limitations to the study. Finally, the Conclusions subsection aims to highlight the main points of our study and draw general conclusions from the investigation.

## Methods

### Electroencephalography Systems

The investigation focused on 7 currently available mobile EEG devices. Table 2 shows the devices and summarizes their characteristics that are briefly described in the following.

NeuroSky’s MindCap device is a 1-channel EEG system. It comes with a frontal electrode and an ear clip reference electrode. The use of conductive gel is not necessary, and the signal is transmitted wirelessly through Bluetooth interface. The weight is 119 g. The device is recommended for neurofeedback training and gaming.

Emotiv’s EPOC device comes with 14 saline-based wet felt sensors. These are mounted on quite flexible plastic branches. The signal is transmitted wirelessly through Bluetooth interface. The EPOC device has a weight of 116 g.

**Figure 1:**
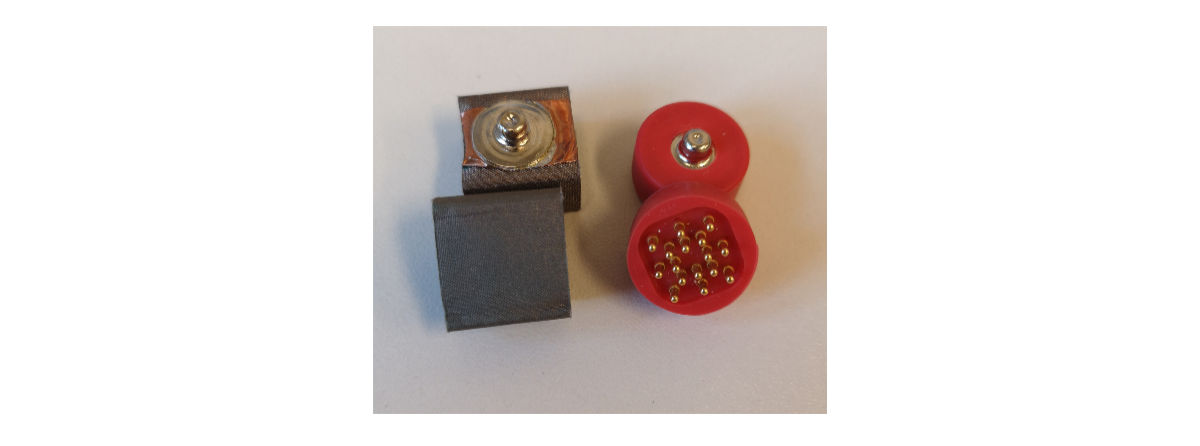
Foam-based frontal electrodes (left) and spring-loaded pin electrodes (right).

Mindo’s 4S Jellyfish device is a wireless dry electrode EEG device. The 4 electrodes that are mounted on a headband can be applied at either frontal or parietal sites. In our case of frontal EEG, foam-based electrodes (Figure 1, left) are recommended. In case of parietal EEG, spring-loaded pin electrodes (Figure 1, right) are to be applied. The reference is an adhesive electrode at the mastoid. The device weighs 95 g.

Mindo’s 32 Trilobite device comprises 32 EEG channels. The frontal 3 of them are foam-based electrodes (Figure 1, left). The remaining 29 are spring-loaded pin electrodes (Figure 1, right). Furthermore, the device includes a ground and a reference electrode, both applied with a clip on the ear lobes. Signal transmission occurs wirelessly through Bluetooth. Its weight is 524 g.

**Figure 2:**
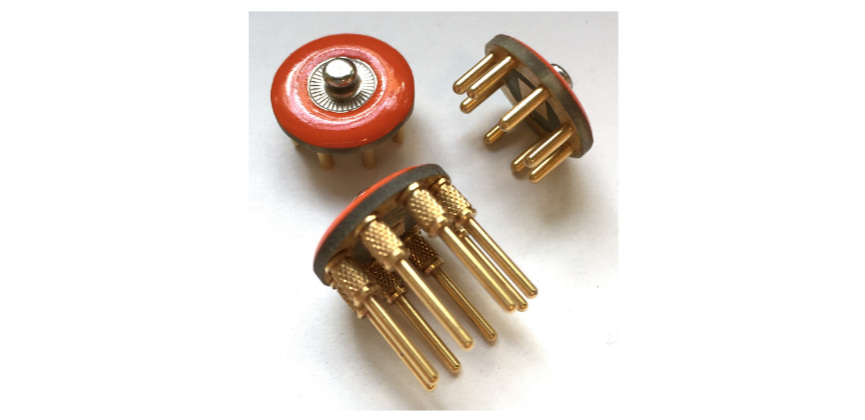
Pin electrodes of g.tec's g.SAHARA device.

BRI’s BR8+ device has got 8 dry electrodes. The frontal 2 of them are foam-based electrodes (Figure 1, left). The remaining 6 are spring-loaded pin electrodes (Figure 1, right). The device includes ground and reference ear clip electrodes and a wireless signal transmission through Bluetooth. The earpads of the device do not have any technical functionality. They are thought to reduce the headset pressure and help positioning the headset at the center of the head. The BR8+ weighs 269 g.

g.tec’s g.SAHARA/g.Nautilus device comprises 16 pin electrodes (Figure 2) that are mounted on a traditional EEG cap. The cap size can vary among small, medium, and large. However, to reduce financial costs, we used only the medium-sized cap. Adhesive ground and reference electrodes are applied at the mastoids. The signal is transmitted wirelessly by means of g.Nautilus device that is attached at the back of the EEG cap. It has a weight of 233 g.

g.tec’s g.LADYbird/g.Nautilus device is a traditional gel-based EEG system with 16 active electrodes. An ear clip electrode serves as reference. Similar to the g.SAHARA/g.Nautilus device, the cap size can vary. However, in our study, we used only the medium-sized cap. The g.Nautilus device at the back of the cap allows for wireless signal transmission. The total weight of the EEG headset amounts to 165 g. Unlike the other devices, the g.LADYbird/g.Nautilus device is not designed for home and biofeedback applications. It is primarily developed for research and medical use and the treatment of locked-in patients. We included it to our study as state-of-the-art reference for EEG regarding user experience issues.

Finally, all manufacturers of our EEG devices promote their EEG systems as highly comfortable and easy to use.

**Table 2:** Electroencephalography (EEG) devices used.

EEG device	Headset	Electrode type	Number of electrodes	Weight
MindCap (NeuroSky Inc, San Jose, CA, USA)	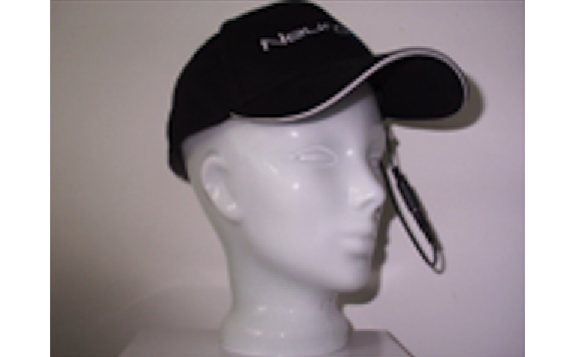	Dry	1	119 g
EPOC (Emotiv Inc, San Francisco, CA, USA)	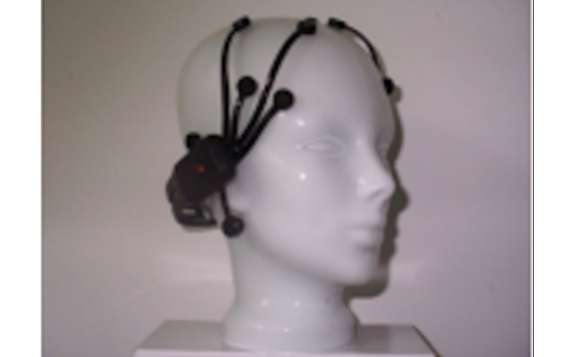	Saline-based	14	116 g
Jellyfish (Mindo, Hsinchu, Taiwan)	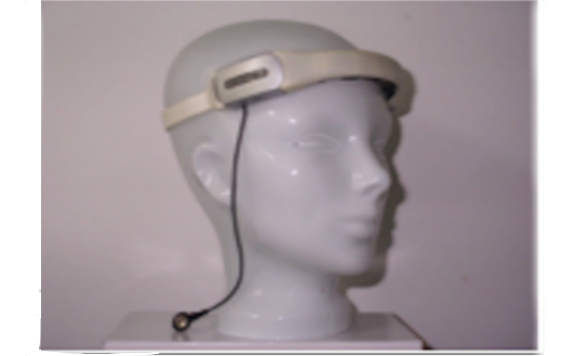	Foam-based	4	95 g
Trilobite (Mindo, Hsinchu, Taiwan)	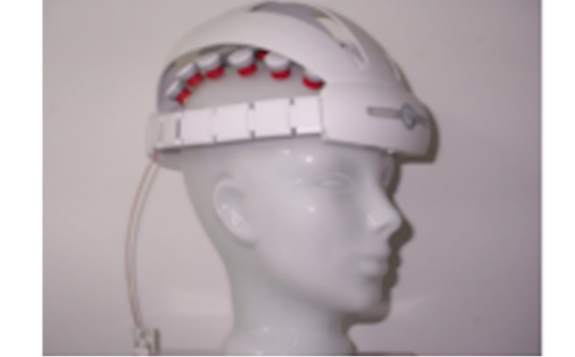	3 foam-based, 29 spring-loaded pins	32	524 g
BR8+ (BRI Inc, Hsinchu, Taiwan)	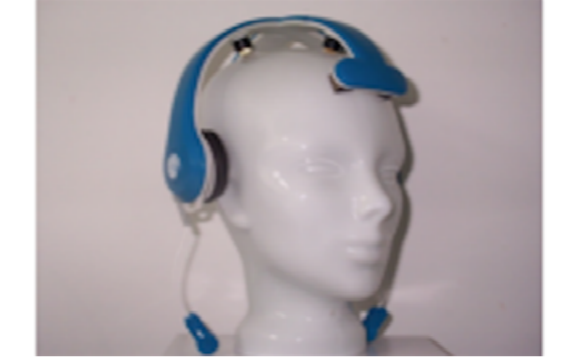	2 foam-based, 6 spring-loaded pins	8	269 g
g.SAHARA (g.tec GmbH, Graz, Austria)	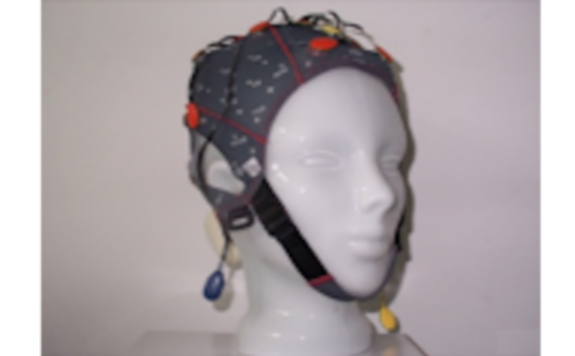	Pin electrodes	16	233 g
g.LADYbird (g.tec GmbH, Graz, Austria)	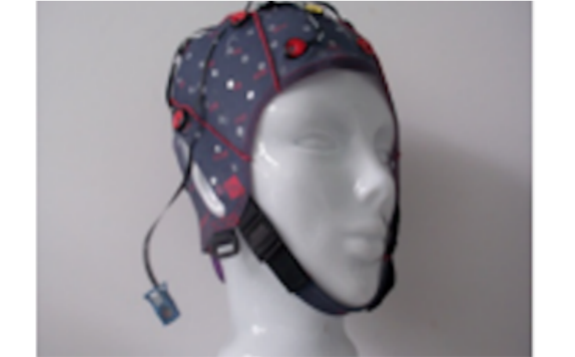	Gel-based	16	165 g

### Procedure and Subjects

Our study took place in a typical office setting. The 24 subjects participating (Table 3) completed over the course of 9 consecutive workdays a total of 9 sessions. The first session was aimed at familiarizing the subjects with the computer tasks and games they had to perform while wearing the EEG devices. In this session, we also assessed subjects’ attitude toward technology by means of the 19 items of the TA-EG questionnaire (TA-EG: translated from the original German title: “Fragebogen zur Technikaffinität - Einstellung zu und Umgang mit elektronischen Geräten”) [19-22]. The items are answered on a 5-point Likert scale (1=fully disagree and 5=fully agree) and address 4 dimensions: technology enthusiasm, competence in handling technology, positive attitude, and negative attitudes toward electronic devices. Subjects with calculated values below the median were assigned to the group of negative attitudes, whereas subjects with values over the median were assigned to the group of positive attitudes toward technology.

**Table:** Sample set used for analysis.

Age (years)	Male, n (%)	Female, n (%)	Total, N
26-34	2 (20)	8 (80)	10 (100)
35-49	3 (50)	3 (50)	6(100)
50-66	8 (100)	0 (0)	8(100)
Total	13	11	24

In the following 7 days, 1 device per day was selected in random order and tested independently of the others. Thereby, the subjects wore the device for 60 min and performed the same sequence of tasks and 1-min rest measurements with eyes closed and eyes opened. The devices were applied by an expert. At the end of each session, they were asked how long they would be able to wear the EEG headset. They indicated their answers on a 5-min steps scale between 0 and 120 min. They also answered questions regarding the device’s design. Next, the subjects applied the device on their own. The expert inspected the signal quality of the EEG and gave instructions for improving it when needed. Moreover, 1-min rest measurements with eyes closed and eyes opened were performed, and thereafter, subjects rated the practicability of the device (Table 4). An exception was made for the g.LADYbird device that could not be taken off, reapplied, and properly used because of the smeared gel that builds conductive bridges. For the g.LADYbird device, we solely skipped the rest measurements.

During the last session, all EEG devices were rated. First, paired comparisons were conducted between every 21 pairs of 2 devices presented. Participants were asked to select the headset that they were willing to wear over a longer period of time or even daily. To avoid reliance on memory, subjects were instructed to reapply each of the 2 presented headsets and decide consciously. A mirror in front of them allowed them to include the visual appearance of the headset in their preference rating. Furthermore, we paid attention to the presentation order of the pairs and proceeded as recommended by Ross [23].

**Figure 3:**
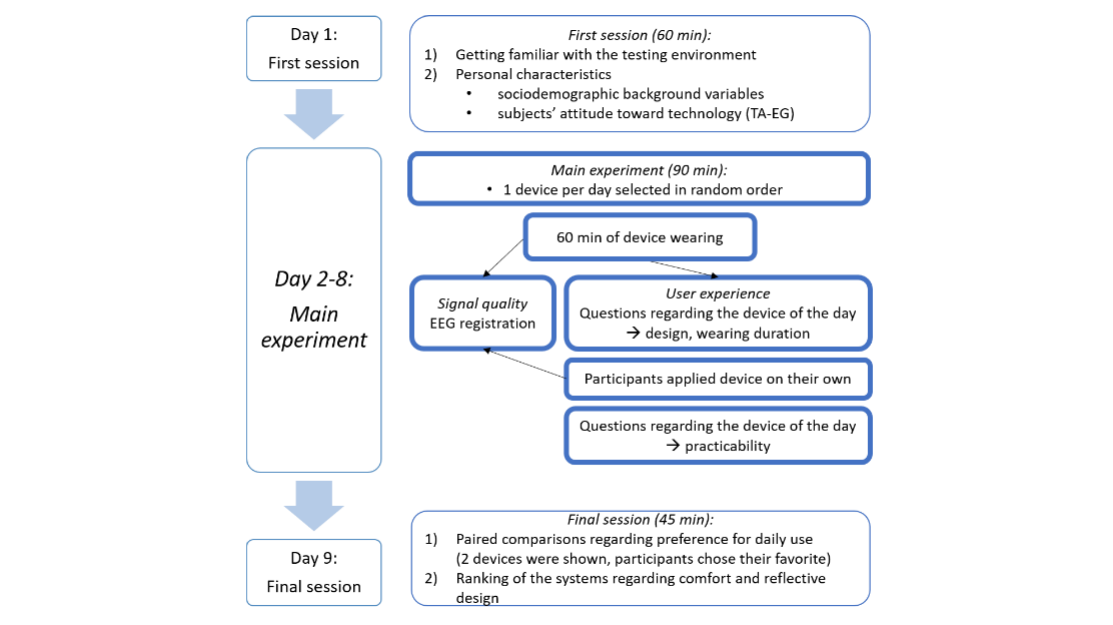
Experimental design of the study. EEG: electroencephalography.

Finally, subjects completed a questionnaire where they had to rank the devices regarding wearing comfort and visual appearance separately (Table 4). Thereby, the item for visual appearance aimed to also integrate aspects from the reflective level of emotional design. Each of the headsets was set on a rank order between 1 (the most appropriate) and 7 (the least appropriate). Figure 3 outlines the experimental design of the study. All procedures were carried out with the adequate understanding and written consent of the subjects. The investigations acquired were approved by the local review board of our institution.

**Table 4:** User experience acquisition.

Aspects of emotional design	Item	Possible answers	Conducted	Research question
Visceral level	The headset has an attractive design	1: does not apply at all and 5: applies fully	After each session	1b
Behavioral level	I could apply and use the EEG^a^ headset without aid	1: does not apply at all and 5: applies fully	After each session	1c
Behavioral level	How long are you able to wear EEG headset? Please mark the maximal-possible time duration in minutes on the scale below	Scale from 0 to 120 with 5 min steps	After each session	1a
Behavioral level	Wearing the device was comfortable	Ranking of the devices: 1: most appropriate and 7: least appropriate	Final session	2
Reflective level	It would not be a problem for me to be seen by my colleagues wearing the device	Ranking of the devices: 1: most appropriate and 7: least appropriate	Final session	2

^a^EEG: electroencephalography.

## Results

### Comparisons Among Devices

The first research objective was concerned with the test of the devices regarding their wearing comfort after a longer period of time, visual appearance, and ease of use. For evaluation, we used subjects’ answers conducted after each session (Table 4). Statistical analysis was conducted using nonparametric Friedman tests of differences among the repeated measures.

### Maximal Possible Wearing Duration Differs Among Devices

Results indicated a highly significant change in maximal possible wearing duration among the EEG devices (χ^2^_6_=40.2, n=24; *P*<.001). Rankings are presented in Table 5. Dunn-Bonferroni posthoc tests were calculated for the examination of the differences among the devices (Table 5; see also Multimedia Appendix 1 for the exact values). Significant differences were obtained between the Trilobite device and all other devices except the BR8+. The Trilobite device was ranked lower regarding maximal wearing duration than the other devices.

### Perception of Headset Design Differs Among Devices

Regarding the visual perception of devices’ headset design, results indicated a significant change in subjects’ ratings (χ^2^_6_=78.7, n=24; *P*<.001). Rankings are presented in Table 6. Dunn-Bonferroni posthoc tests were calculated for the examination of the differences among the devices (Table 6; Multimedia Appendix 2).

**Table 5:** Maximal possible wearing duration (min) for each device over all subjects.

EEG device	Mean (SD)	Median (min, max)
MindCap	92.29 (35.87)	112.5 (5, 120)
Jellyfish	86.66(31.78)	90.0 (30, 120)
BR8+	73.54 (30.16)	60.0 (30, 120)
EPOC	101.87 (25.10)	117.5 (30, 120)
g.Sahara	81.04 (33.45)	80.0 (10, 120)
Trilobite	48.75 (28.59)	50.0 (5, 120)
g.Ladybird	100.41 (23.99)	112.5 (45, 120)

**Table 6:** “The headset has an attractive design.” (1: does not apply at all and 5: applies fully). Statistics calculated over all, male, and female subjects for each device.

EEG device	All		Male		Female	
	Mean (SD)	Median (min, max)	Mean (SD)	Median (min, max)	Mean (SD)	Median (min, max)
MindCap	3.71 (0.95)	4.0 (1, 5)	4.15 (0.68)	4.0 (3, 5)	3.18 (0.98)	3.0 (1, 4)
Jellyfish	3.58 (0.97)	4.0 (2, 5)	3.85 (0.80)	4.0 (2, 5)	3.27 (1.10)	4.0 (2, 5)
BR8+	3.58 (0.97)	4.0 (1, 5)	3.92 (0.64)	4.0 (3, 5)	3.18 (1.16)	3.0 (1, 5)
EPOC	4.08 (0.77)	4.0 (2, 5)	4.23 (0.92)	4.0 (2, 5)	3.91 (0.53)	4.0 (3, 5)
g.Sahara	2.21 (1.10)	2.0 (1, 5)	2.62 (1.12)	3.0 (1, 5)	1.73 (0.90)	2.0 (1, 4)
Trilobite	2.58 (0.92)	2.5 (1, 5)	2.92 (0.86)	3.0 (2, 5)	2.18 (0.87)	2.0 (1, 4)
g.Ladybird	2.08 (0.83)	2.0 (1, 4)	2.46 (0.66)	2.0 (2, 4)	1.64 (0.80)	1.0 (1, 3)

Significant differences were obtained between the g.LADYbird device and all other devices except g.SAHARA and Trilobite. The g.SAHARA device showed significant differences to all devices except Trilobite and g.LADYbird. The Trilobite device showed significant differences to the EPOC, MindCap, and Jellyfish devices. At this point, we also looked at possible gender effects relating to the perception of headsets’ design. We evaluated the ratings separately for male and female participants (Table 6) and found highly significant differences among devices for both groups (male: χ^2^_6_=41.9, n=13, *P*<.001; female: χ^2^_6_=38.3, n=11, *P*<.001). Dunn-Bonferroni posthoc tests for male participants’ ratings indicated significant differences between the Trilobite and EPOC devices as well as between g.SAHARA and MindCap and g.SAHARA and EPOC (Table 6; Multimedia Appendix 3). Furthermore, there were significant differences between the g.LADYbird device and all other devices except g.SAHARA and Trilobite. Dunn-Bonferroni posthoc tests for female participants’ ratings indicated significant differences between the Trilobite and EPOC devices, g.SAHARA and EPOC as well as between g.LADYbird and EPOC and g.LADYbird and Jellyfish (Table 6; Multimedia Appendix 4).

### Practicability Differs Among Devices

Results of subjects’ ratings regarding the practicability of the devices indicated highly significant differences among the EEG devices (χ^2^_6_=83.2, n=24; *P*<.001). Rankings are presented in Table 7. Dunn-Bonferroni posthoc tests were calculated for the examination of the differences among the devices (Table 7; Multimedia Appendix 5).

Significant differences were obtained between the g.LADYbird device and all remaining devices. To evaluate possible differences among subjects related to their attitude toward technology, we used the results from the TA-EG questionnaire and clustered our subjects in 2 groups. Subjects with a value below the overall median of 69.5 (range between 41 and 81) were assigned to the group with a negative attitude toward technology (mean age of cluster: 41 years, 5 females, and 7 males) and subjects with a value over the median to the group with a positive attitude (mean age of cluster: 44 years, 6 females, and 6 males). We evaluated the practicability ratings separately and found highly significant differences among devices for both groups (negative attitude: χ^2^_6_=48.5, n=12, *P*<.001; positive attitude: χ^2^_6_=40.6, n=12, *P*<.001).

Dunn-Bonferroni posthoc tests for the ratings of subjects with a negative attitude toward technology indicated significant differences between the g.LADYbird and all remaining devices (Table 7; Multimedia Appendix 6). Dunn-Bonferroni posthoc tests for the ratings of subjects with a positive attitude toward technology indicated significant differences between the g.LADYbird and all other devices except the Trilobite and g.SAHARA (Table 7; Multimedia Appendix 7).

The critical reader could argue that for evaluating the practicability, the signal quality of the device had to be taken into account after self-fitting the device. For the sake of completeness, we compared the signal quality of the rest measurements from self-fitting versus expert fitting of the system. The evaluation of the electroencephalogram was done in the time domain manually. A medical technical assistant with specialization in EEG and years of experience visually inspected the electroencephalograms and manually marked artifact segments. We computed the percentage of denoted artifacts compared with the entire recording time for each channel. We calculated the means over the channels for each subject and device. For comparison between the signal qualities from self-fitting versus expert fitting, we conducted a Wilcoxon paired difference test for each EEG system. The results are presented in Table 8. Rest measurements with closed eyes did not show significant differences between the fittings for none of the devices. Rest measurements with eyes opened indicated significant differences between the fittings for the BR8+ and the g.SAHARA devices (BR8+: z=−3.886, *P*<.001, r=0.56; g.SAHARA: z=4:086, *P*<.001, r=0.59).

For readers more interested in the signal quality evaluation of the devices, we would like to draw their attention on our paper on that topic [24].

**Table 7:** “I could apply and use the EEG headset without aid.” (1: does not apply at all and 5: applies fully). Statistics calculated over all subjects, subjects with positive attitude, and subjects with negative attitude toward technology for each device. EEG: electroencephalography.

EEG device	All		Positive attitude		Negative attitude	
	Mean (SD)	Median (min, max)	Mean (SD)	Median (min, max)	Mean (SD)	Median (min, max)
MindCap	4.63 (0.64)	5.0 (3, 5)	4.42 (0.79)	5.0 (3, 5)	4.42 (0.79)	5.0 (3, 5)
Jellyfish	4.67 (0.56)	5.0 (3, 5)	4.50 (0.67)	5.0 (3, 5)	4.50 (0.67)	5.0 (3, 5)
BR8+	4.21 (1.10)	5.0 (2, 5)	3.83 (1.19)	4.0 (2, 5)	3.83 (1.19)	4.0 (2, 5)
EPOC	4.54 (0.58)	5.0 (3, 5)	4.50 (0.52)	4.5 (4, 5)	4.50 (0.52)	4.5 (4, 5)
g.Sahara	4.04 (1.04)	4.0 (2, 5)	3.58 (1.24)	4.0 (2, 5)	3.58 (1.24)	4.0 (2, 5)
Trilobite	3.54 (1.31)	4.0 (1, 5)	3.00 (1.27)	3.0 (1, 5)	3.00 (1.27)	3.0 (1, 5)
g.Ladybird	1.75 (0.89)	1.5 (1, 5)	1.75 (0.86)	1.5 (1, 3)	1.75 (0.86)	1.5 (1, 3)

**Table 8:** Artifact proportions (%) of rest measurements with eyes open and closed from self-fitting and expert fitting of the system averaged over channels and subjects and considered for each device separately.

EEG device	Eyes closed				Eyes open			
	Expert fitting		Self-fitting		Expert fitting		Self-fitting	
	Mean (SD)	Median (min, max)	Mean (SD)	Median (min, max)	Mean (SD)	Median (min, max)	Mean (SD)	Median (min, max)
MindCap	15.37 (33.54)	0.0 (0.0, 99.9)	17.60 (35.92)	0.0 (0.0, 99.9)	16.75 (33.38)	0.0 (0.0, 99.9)	10.77 (23.68)	0.0 (0.0, 99.9)
Jellyfish	23.26 (27.56)	13.4 (.0, 99.7)	14.64 (18.16)	6.9 (0.0, 61.9)	24.15 (25.98)	14.7 (0.0, 87.3)	20.94 (22.88)	11.8 (0.0, 80.0)
BR8+	48.38 (21.55)	49.9 (3.7, 87.5)	59.51 (22.44)	63.23 (12.5, 99.9)	45.12 (17.36)	47.7 (14.3, 80.0)	75.62 (20.89)	78.4 (26.5, 100)
EPOC	23.25 (37.07)	3.6 (0.0, 99.9)	37.82 (45.16)	11.4 (0.0, 99.9)	22.18 (36.61)	5.0 (0.0, 99.9)	37.60 (42.69)	13.3 (0.0, 99.9)
g.Sahara	32.05 (11.47)	34.1 (5.7, 55.5)	32.54 (13.46)	33.0 (0.0, 65.1)	9.79 (12.74)	4.0 (0.0, 41.8)	21.33 (16.85)	18.5 (3.5, 74.5)
Trilobite	29.14 (26.33)	18.6 (3.1, 106.25)	22.10 (26.19)	14.4 (0.0, 106.1)	33.83 (25.32)	23.9 (0.0, 91.4)	23.69 (20.56)	16.6 (0.0, 83.5)

### Wearing Comfort and Visual Appearance

Our research question 2 asked if visual appearance affects the overall rating of the devices more than their wearing comfort. For the evaluation, we used multiple linear regression analysis. Ranking values of the items for visual appearance and wearing comfort (Table 4) served as independent variables. The criterion was the devices’ ranking order regarding preference for daily use. This was calculated from the conducted paired comparisons.

For the sake of completeness, we have to mention that results from paired comparisons were not transitive for 6 subjects. In these cases, some devices have been selected with the same frequency, and thus, subjects’ preference could not be mapped on an ordinal scale. Analysis of these subjects’ decisions regarding the less rejected devices did not yield to a result, either. Hence, the 6 subjects with inconsistent answers were disclosed from further analysis.

We computed a multiple linear regression for each device separately. The results are presented in Table 9. Wearing comfort and visual appearance of the devices were able to statistically significant predict subjects’ preference for daily use, except for the g.LADYbird device (*F*_2,15_=0.752; *P*=.49). Wearing comfort had a large impact on device preference for almost all devices, whereas visual appearance was a poor predictor. An exception was the EPOC device. Hereby, visual appearance had a large impact on the preference, whereas wearing comfort had none. For the BR8+ device, both predictors were important. However, the wearing comfort was more influential.

At this point, we also looked at possible gender effects relating to the utilitarian versus hedonic aspects of the experience. For the male participants, wearing comfort and visual appearance were able to statistically significant predict subjects’ preference for daily use, except for the g.LADYbird device (*F*_2,7_=0.147; *P*=.87). Wearing comfort had a large impact on device preference for all devices except for the EPOC device where visual appearance was a better predictor. For the female participants, a significant regression equation with significant predictors was found for the Jellyfish (*F*_2,5_=29.837; *P*=.002) and EPOC (*F*_2,5_=25.571, *P*=.002) devices. For Jellyfish, wearing comfort significantly predicted subjects’ preference, whereas for EPOC, visual appearance had a greater impact on subjects’ preference ratings. Overall, it can be said that in cases where the regression models became significant, we were not able to identify opposing effects between female and male participants (Table 9).

**Table 9:** Results of multiple linear regression analysis for each device.

EEG^a^ device and gender		R^2^	Model		Wearing comfort		Visual appearance	
			*F* test (*df*)	*P* value	Coefficient	*P* value	Coefficient	*P* value
**MindCap**								
	Both	0.938	112.518 (2,15)	<.001	1.112	<.001	−0.245	.21
	Male	0.989	305.051 (2,7)	<.001	0.985	<.001	−0.024	.90
	Female	0.707	6.018 (2,5)	.05	0.472	.16	0.067	.79
**Jellyfish**							
	Both	0.825	35.319 (2,15)	<.001	0.797	<.001	0.069	.77
	Male	0.764	11.357 (2,7)	.006	0.751	.01	0.210	.60
	Female	0.923	29.837 (2,5)	.002	0.731	.008	0.235	.59
**BR8+**							
	Both	0.846	41.182 (2,15)	<.001	0.701	<.001	0.327	.04
	Male	0.952	70.150 (2,7)	<.001	0.802	.001	0.312	.07
	Female	0.498	2.479 (2,5)	.18	0.540	.09	−0.010	.98
**EPOC**							
	Both	0.849	42.080 (2,15)	<.001	0.149	.14	0.656	<.001
	Male	0.823	16.286 (2,7)	.002	0.191	.53	0.627	.02
	Female	0.911	25.571 (2,5)	.002	0.153	.14	0.655	.009
**g.SAHARA**							
	Both	0.742	21.603 (2,15)	<.001	0.740	<.001	−0.040	.76
	Male	0.939	54.275 (2,7)	<.001	0.777	<.001	0.011	.89
	Female	0.633	4.312 (2,5)	.08	0.677	.03	0.000	>.99
**Trilobite**							
	Both	0.737	21.026 (2,15)	<.001	0.943	<.001	0.139	.39
	Male	0.770	11.706 (2,7)	.006	1.043	.002	0.109	.63
	Female	0.485	2.354 (2,5)	.19	0.620	.12	0.260	.41
**g.LADYbird**							
	Both	0.091	0.752 (2,15)	.49	0.018	.93	0.243	.25
	Male	0.040	0.147 (2,7)	.87	0.063	.68	0.038	.75
	Female	0.335	1.261 (2,5)	.36	−0.400	.42	2.300	.20

^a^EEG: electroencephalography.

## Discussion

### Comparisons Among Devices

In our first research objective, we were concerned to test the devices regarding 3 user experience aspects: wearing comfort, visual appearance, and ease of use.

### Pin Electrodes Had the Lowest Wearing Comfort

Evaluation of the maximal possible wearing time as an indicator of devices’ wearing comfort revealed the Trilobite device to be significantly less pleasant to wear than the remaining. The reason could be the uncomfortable pin electrodes. Overall means of maximal possible wearing duration indicated devices without pin electrodes such as the EPOC, MindCap, and g.LADYbird as the most favorable for a longer wearing time and with significant differences to the Trilobite. The finding that pin electrodes were less preferred was similar to findings by Grozea et al [14] but inconsistent to the results by Nijboer et al [18] and Izdebski et al [16]. However, Hairston et al [17] also emphasized the importance of the headset’s ability to adjust to the different heads to assure comfort. In their work, they highlighted the need of flexible headsets to assure comfort during wearing. This aspect was also prominent in the work of Izdebski et al [16] who found that cap fit was rated as poor for headsets with rigid headsets. In our study, Trilobite’s headset was the most rigid one. Furthermore, the Trilobite device was much heavier than the other devices. These 2 facts could have multiplied the impact of the pin electrodes on wearing comfort. The BR8+ device had pin electrodes, a rather rigid headset but less weight. Similar to the Trilobite, it yielded small values regarding the maximal possible wearing duration. The g.SAHARA with pin electrodes but flexible headset and less weight had small wearing duration ratings, but these were higher than those of the Trilobite and BR8+ devices. We concluded that pin electrodes had the lowest wearing comfort, in particular when coupled with a rigid, heavy headset.

### An Unobtrusive Design Coped Better With Individual Preferences

Headset design is not only responsible for the wearing comfort but also primarily responsible for device’s visual appearance. Overall ratings of headset design indicated that the devices with a traditional EEG cap (ie, g.LADYbird and g.SAHARA) were significantly less preferred than all others, except the Trilobite device. The latter was also significantly less preferred than the MindCap, Jellyfish, and EPOC devices. Females’ ratings indicated more variability than males’ ratings leading to less significant differences among the devices. However, both genders perceived the design of g.LADYbird’s and g.SAHARA’s traditional caps and Trilobite’s helmet as less attractive. Both groups primarily preferred the headsets of EPOC and Jellyfish with EPOC, indicating more significant differences to the other devices, in particular, by female subjects. This result was consistent with the results by Nijboer et al [18] where participants rated their appearance with the EPOC as best. Nijboer et al stated that reasons for the refusal of caps were that the whole head and part of the face were covered, and hair was flattened and invisible. In our study, the g.LADYbird, g.SAHARA, Trilobite, and MindCap devices covered subjects’ whole head. However, ratings of the MindCap were significantly better compared with the other 3 devices. This was particularly true among the male subjects. We assumed that rating of the design was related to aspects of aesthetics, fashion style, and individual preference. These aspects might be strongly connected to the reflective level of emotional design. An unobtrusive headset design could have more potential to cope with different individual preferences because it is not eye-catching.

### Practicability Was Closely Linked to Gel Electrodes and Attitude Toward Technology

**Figure 4:**
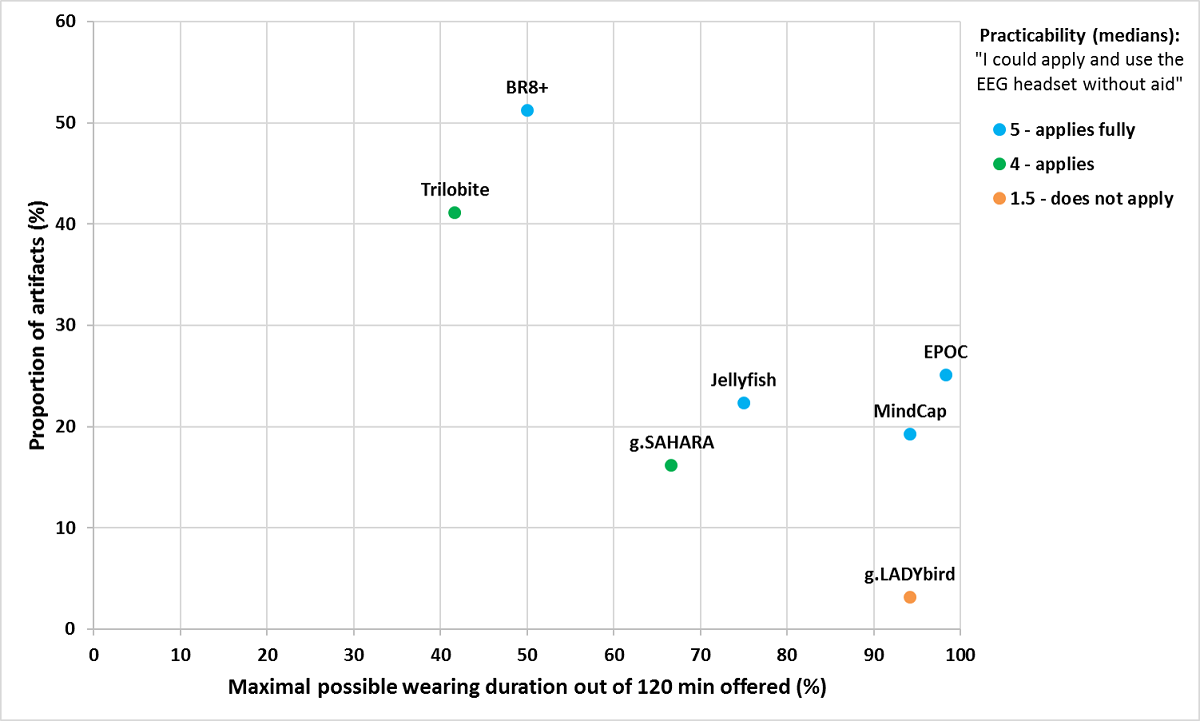
Relation between signal quality and comfort and trade-off against practicability. EEG: electroencephalography.

Finally, we asked the subjects to rate the ease of use of the devices. Results indicated significant differences between the gel-based g.LADYbird and all remaining devices. This was reasonable, especially when considering that a second person was needed for applying the gel. Furthermore, subjects had to wash their hair after they took off the cap. We concluded that the effort for use was definitely high. The g.SAHARA and Trilobite devices were also rated as less easy to use. We supposed that this might be because of their larger number of electrodes but have to be aware that g.SAHARA had only 2 electrodes more than the EPOC device. Subjects with a negative attitude toward technology showed similar results regarding the practicability of the devices. However, subjects with a positive attitude toward technology did not indicate significant differences between the gel-based g.LADYbird and pin-based g.SAHARA neither between the g.LADYbird and Trilobite devices. Although these findings were surprising, we supposed that technical affine subjects were more critical during their ratings, and this could lead to more variability in their ratings. Taken the results of the signal quality comparison (Figure 4) into account, we noted similar tendencies between practicability ratings from subjects with a positive attitude toward technology and increased proportion of artifacts by self-fitting the devices. This was particularly true during the rest measurements with eyes opened, as subjects might have behaved more actively than with eyes closed. Thereby, the BR8+, g.SAHARA, and, to a lesser extent, the EPOC devices yielded more artifacts when compared with the fittings by an expert and revealed less practicability when rated by technical affine subjects. Nevertheless, the g.LADYbird device had the worst practicability ratings across subjects although a limitation of our study might be that we did not give the opportunity to the subjects to apply the device and the gel on their own. We believe that self-fitting of the gel-based electrodes would not have altered the ratings but must admit that future user experience research should consider this issue. Finally, we argue that subjects with a positive attitude toward technology were more accurate in their rating of device practicability.

In conclusion, although the practicability of the devices was closely linked to gel or dry electrodes, wearing comfort and design of the devices seemed to be more expressive. Thereby, we observed that devices that could be worn for a longer period of time did not always have an attractive design.

### Wearing Comfort and Visual Appearance

In our second research question, we were interested to find out if wearing comfort was more important to the user than the visual appearance of the device. Thus, we asked subjects to rank all devices regarding both aspects separately. Furthermore, paired comparisons of the devices led us to a rank order regarding preference for daily use.

Results of a multiple linear regression analysis for each device indicated that, in general, wearing comfort was the better predictor for users’ device preference. Exceptions were the EPOC and the g.LADYbird devices. Although for the g.LADYbird, none of the 2 aspects seemed to have any impact on device’s preference ranking; the results for the EPOC device revealed an opposite tendency, that is, EPOC’s visual appearance influenced subject’s decision more than its wearing comfort. A reason for this could be that EPOC’s wearing comfort was unobtrusive although its design was futuristic and professional. We assumed that this attracted the subjects and gave more weight to the visual appearance when it came to a preference for daily use. Interestingly, the design of the BR8+ was also one of the most modern and futuristic ones. The fact that BR8+’s visual appearance was a supplementary predictor to its comfort seemed to confirm our assumption.

Regarding the results of the g.LADYbird device, we had to speculate. The device was assumed to not cause any head pressure; hence, wearing comfort should be unobtrusive and a weak predictor for the preference for daily use. Its visual appearance was indeed not very attractive for daily wearing. However, this fact did not have a large influence on the preference either, similar to the g.SAHARA device that had the same cap. The main difference to all other devices was the application of gel and the necessity to wash the hair after each use of the device. Although comfortable to wear, the gel-based electrodes were undoubtedly inconvenient for daily use outside the laboratory. Hence, the ease of use could have affected the preference more than the examined factors.

Male and female participants did not show opposing results related to the predictors of daily use preference. Although almost all models (except g.LADYbird) became significant for the male participants, for the female participants, only 2 models reached the significance level (Jellyfish and EPOC). An explanation could be that females’ ratings were not as consistent as males’ ratings among each other. However, we have to be also aware of the small number of participants (8 females vs 10 males) that could have led to this result. To explore gender differences related to utilitarian versus hedonic aspects of experience, more research with larger subsets is needed. We have to draw attention to our sample’s structure (Table 3) consisting of young female and older male participants. Disentangle the gender and age factors at these numbers seemed not possible. We assumed that regarding emotional design, the gender factor is more influential than the age, but the reader should note that the latter could have an effect, too. Further research should emphasize on this issue.

In general, the results of both genders emphasized that visual appearance was a better predictor only for the EPOC device. By taking into account the reflective level of emotional design, we add new insight about how the factors of comfort and visual appearance translate to user preference. Our results broaden the assumption by Nijboer et al [18] who postulated that the preference of EPOC was an evidence for the fact that aesthetics might be more important than comfort.

### Conclusions

In our study, we investigated the user experience of mobile EEG devices. We compared 7 different EEG devices and offered a differentiated look at emerging mobile and gel-free EEG technology. The results yielded are summarized in Table 10. For the sake of convenience, we report only the artifact proportion differences between self-fitting and expert fitting from the eyes-closed measurement.

In addition, we gave insight into the relation between user experience aspects and device preference. The wearing comfort given by a device was the main factor for its daily use. The visual appearance of the device was certainly an important point. However, it only became influential when comfort was assured. Users were not willing to accept less comfort for a more attractive headset design. The reflective level of emotional design became important only if the behavioral level of the product was satisfactory.

To provide practical information to users of EEG devices, we combined the signal quality results from the study by Radüntz [24] with the current user experience results and concluded which system could be used under which condition. The EPOC device achieved the best results regarding user experience, but it suffered from a large proportion of artifacts. Although the EPOC device can be used in public because of its attractive design and the feeling of ease of use, potential users should be aware of the issues regarding signal quality, in particular, if the device is self-applied by a layman. Outstanding performances regarding maximal possible wearing duration and signal quality were obtained for the traditional gel-based but mobile g.LADYbird device. This device can be recommended for neuroscience research where precise and prolonged measurements are required without any deductions in comfort. However, devices wearing in public and self-application are not recommended. The MindCap device reviled good user experience results and satisfying signal quality. Users must consider that scientifically valid assertions could be hampered because of only 1 electrode available. The Jellyfish and g.SAHARA devices yielded similar results regarding comfort but differences regarding design (ie, better results for Jellyfish) and signal quality (ie, better results for g.SAHARA). We believe that g.SAHARA is a good solution for field experiments, where subjects are not exposed to the general public, and signal quality is important. Nevertheless, researchers should be aware of potential comfort issues that could arise in the course of time because of the pin electrodes. Potential applications for the Jellyfish device might be better suited for the gaming or biofeedback sector. The BR8+ and Trilobite devices did not meet our requirement for user experience, in particular, because of comfort issues. Furthermore, signal quality was lacking. Figure 4 illustrates the trade-offs between signal quality and user experience so that readers might be able to see if there are any devices of sufficient quality that might also be acceptable for daily use. The x-axis depicts devices’ comfort rankings, calculated as a percentage of the maximal possible wearing duration in minutes out of 120 min offered. The y-axis represents the proportion of artifacts taken from the study by Radüntz [24].

Finally, we have to admit that there might be further factors that could have contributed to the preference decision. Our research could be seen as a precondition for the use of emerging EEG technology under realistic conditions in field experiments with longer duration. It paves the way for the development of usable applications with wearables and contributes to consumer health informatics and health-enabling technologies. Furthermore, our results provided guidance for the technological development direction of new EEG devices related to aspects of emotional design.

It has to be mentioned that the EEG equipment market shows rapid development. During this study, new devices appeared on the market that could not be tested, for example, the actiCAP Xpress Twist/LiveAmp device by Brain Products or the highly innovative approach using in-ear EEG technology [25,26]. However, our study design could easily be used in subsequent studies of new devices and benchmark the evaluation of further emerging EEG technology. Integration of test results from new devices into the findings already in existence would make it possible to compare the user experience of emerging EEG technology.

**Table 10:** User experience results of tested electroencephalography devices (medians over all subjects).

EEG^a^ device	Comfort: maximal wearing duration (min)	Design (higher values indicate a more attractive design)	Practicability (higher values indicate greater practicability)	Artifact proportions (eyes closed: self-fitting-expert fitting [%]; higher values indicate more artifacts when self-fitted)
MindCap	113	4	5	2.2
Jellyfish	90	4	5	−8.6
BR8+	60	4	5	11.1
EPOC	118	4	5	14.6
g.SAHARA	80	2	4	0.5
Trilobite	50	2.5	4	−7
g.LADYbird	113	2	1.5	Not applicable

^a^EEG: electroencephalography.

## Appendix

Multimedia Appendix 1 Maximal possible wearing duration for each device over all subjects.

Multimedia Appendix 2 Attractive design ratings for each device over all subjects.

Multimedia Appendix 3 Attractive design ratings for each device over the male subjects.

Multimedia Appendix 4 Attractive design ratings for each device over the female subjects.

Multimedia Appendix 5 Practicability ratings for each device over all subjects.

Multimedia Appendix 6 Practicability ratings for each device over subjects with negative attitude toward technology.

Multimedia Appendix 7 Practicability ratings for each device over subjects with positive attitude toward technology.

## Abbreviation

EEG: Electroencephalography
