# Supplementary material for: User Experience of 7 Mobile Electroencephalography Devices: Comparative Study
Source: JMIR Mhealth Uhealth. 2019 Sep 3;7(9):e14474. doi: 10.2196/14474 (PMC6751099; doi:10.2196/14474)
Supplement: Multimedia Appendix 1 [file mhealth_v7i9e14474_app1.pdf]

Multimedia Appendix 1

Appendix with the results of Dunn-Bonferroni post-hoc tests for the examination of the differences between the devices:

Maximal-possible wearing duration for each device over all subjects (N=24)

Pairwise Comparisons

Sample 1-Sample 2	Test Statistic	Std. Error	Std. Test Statistic	Sig.	Adj. Sig. (P)	Effect size (r)
Trilobite-BR8+	1.375	.624	2.205	.03	.58	0.17
Trilobite-g.SAHARA	2.000	.624	3.207	.001	.03	0.25
Trilobite-Jellyfish	2.042	.624	3.274	.001	.02	0.25
Trilobite-MindCap	2.563	.624	4.109	<.001	.001	0.32
Trilobite-g.LADYbird	-2.771	.624	-4.443	<.001	<.001	0.34
Trilobite-EPOC	3.250	.624	5.212	<.001	<.001	0.40
BR8+-g.SAHARA	-.625	.624	-1.002	.32	1.00	0.08
BR8+-Jellyfish	.667	.624	1.069	.29	1.00	0.08
BR8+-MindCap	1.188	.624	1.904	.06	1.00	0.15
BR8+-g.LADYbird	-1.396	.624	-2.238	.03	.53	0.17
BR8+-EPOC	-1.875	.624	-3.007	.003	.06	0.23
g.SAHARA-Jellyfish	.042	.624	.067	.95	1.00	0.01
g.SAHARA-MindCap	.563	.624	.902	.37	1.00	0.07
g.SAHARA-g.LADYbird	-.771	.624	-1.236	.22	1.00	0.10
g.SAHARA-EPOC	1.250	.624	2.004	.05	.95	0.15
Jellyfish-MindCap	.521	.624	.835	.40	1.00	0.06
Jellyfish-g.LADYbird	-.729	.624	-1.169	.24	1.00	0.09
Jellyfish-EPOC	-1.208	.624	-1.938	.05	1.00	0.15
MindCap-g.LADYbird	-.208	.624	-.334	.74	1.00	0.03
MindCap-EPOC	-.688	.624	-1.102	.27	1.00	0.09
g.LADYbird-EPOC	.479	.624	.768	.44	1.00	0.06

Each row tests the null hypothesis that the Sample 1 and Sample 2 distributions are the same.

Asymptotic significances (2-sided tests) are displayed. The significance level is .05.
