# Supplementary material for: User Experience of 7 Mobile Electroencephalography Devices: Comparative Study
Source: JMIR Mhealth Uhealth. 2019 Sep 3;7(9):e14474. doi: 10.2196/14474 (PMC6751099; doi:10.2196/14474)
Supplement: Multimedia Appendix 2 [file mhealth_v7i9e14474_app2.pdf]

Multimedia Appendix 2

Appendix with the results of Dunn-Bonferroni post-hoc tests for the examination of the differences between the devices:

Attractive design ratings for each device over all subjects (N=24)

Pairwise Comparisons

Sample 1-Sample 2	Test Statistic	Std. Error	Std. Test Statistic	Sig.	Adj. Sig. (P)	Effect size (r)
g.LADYbird-g.SAHARA	.333	.624	.535	.59	1.00	0.04
g.LADYbird-Trilobite	.729	.624	1.169	.24	1.00	0.09
g.LADYbird-BR8+	2.521	.624	4.042	<.001	.001	0.31
g.LADYbird-Jellyfish	2.792	.624	4.477	<.001	<.001	0.35
g.LADYbird-MindCap	2.958	.624	4.744	<.001	<.001	0.37
g.LADYbird-EPOC	3.646	.624	5.846	<.001	<.001	0.45
g.SAHARA-Trilobite	-.396	.624	-.635	.53	1.00	0.05
g.SAHARA-BR8+	2.188	.624	3.508	<.001	.01	0.27
g.SAHARA-Jellyfish	2.458	.624	3.942	<.001	.002	0.30
g.SAHARA-MindCap	2.625	.624	4.209	<.001	.001	0.32
g.SAHARA-EPOC	3.313	.624	5.312	<.001	<.001	0.41
Trilobite-BR8+	1.792	.624	2.873	.004	.09	0.22
Trilobite-Jellyfish	2.063	.624	3.307	.001	.02	0.26
Trilobite-MindCap	2.229	.624	3.575	<.001	.007	0.28
Trilobite-EPOC	2.917	.624	4.677	<.001	<.001	0.36
BR8+-Jellyfish	.271	.624	.434	.66	1.00	0.03
BR8+-MindCap	.438	.624	.702	.48	1.00	0.05
BR8+-EPOC	-1.125	.624	-1.804	.07	1.00	0.14
Jellyfish-MindCap	.167	.624	.267	.79	1.00	0.02
Jellyfish-EPOC	-.854	.624	-1.370	.17	1.00	0.11
MindCap-EPOC	-.688	.624	-1.102	.27	1.00	0.09

Each row tests the null hypothesis that the Sample 1 and Sample 2 distributions are the same.

Asymptotic significances (2-sided tests) are displayed. The significance level is .05.
