# Supplementary material for: User Experience of 7 Mobile Electroencephalography Devices: Comparative Study
Source: JMIR Mhealth Uhealth. 2019 Sep 3;7(9):e14474. doi: 10.2196/14474 (PMC6751099; doi:10.2196/14474)
Supplement: Multimedia Appendix 3 [file mhealth_v7i9e14474_app3.pdf]

Multimedia Appendix 3

Appendix with the results of Dunn-Bonferroni post-hoc tests for the examination of the differences between the devices:

Attractive design ratings for each device over the male subjects (N=13)

Pairwise Comparisons

Sample 1-Sample 2	Test Statistic	Std. Error	Std. Test Statistic	Sig.	Adj. Sig. (P)	Effect size (r)
g.LADYbird-g.SAHARA	.385	.847	.454	.65	1.00	0.05
g.LADYbird-Trilobite	.846	.847	.999	.32	1.00	0.10
g.LADYbird-Jellyfish	2.654	.847	3.132	.002	.04	0.33
g.LADYbird-BR8+	2.769	.847	3.268	.001	.02	0.34
g.LADYbird-MindCap	3.308	.847	3.904	<.001	.002	0.41
g.LADYbird-EPOC	3.500	.847	4.131	<.001	.001	0.43
g.SAHARA-Trilobite	-.462	.847	-.545	.57	1.00	0.06
g.SAHARA-Jellyfish	2.269	.847	2.678	.007	.16	0.28
g.SAHARA-BR8+	2.385	.847	2.814	.005	.10	0.29
g.SAHARA-MindCap	2.923	.847	3.450	.001	.01	0.36
g.SAHARA-EPOC	3.115	.847	3.677	<.001	.005	0.39
Trilobite-Jellyfish	1.808	.847	2.133	.03	.69	0.22
Trilobite-BR8+	1.923	.847	2.270	.02	.49	0.24
Trilobite-MindCap	2.462	.847	2.905	.004	.08	0.30
Trilobite-EPOC	2.654	.847	3.132	.002	.04	0.33
Jellyfish-BR8+	-.115	.847	-.136	.89	1.00	0.01
Jellyfish-MindCap	.654	.847	.772	.44	1.00	0.08
Jellyfish-EPOC	-.846	.847	-.999	.32	1.00	0.10
BR8+-MindCap	.538	.847	.635	.53	1.00	0.07
BR8+-EPOC	-.731	.847	-.862	.39	1.00	0.09
MindCap-EPOC	-.192	.847	-.227	.82	1.00	0.02

Each row tests the null hypothesis that the Sample 1 and Sample 2 distributions are the same.

Asymptotic significances (2-sided tests) are displayed. The significance level is .05.
