# Supplementary material for: User Experience of 7 Mobile Electroencephalography Devices: Comparative Study
Source: JMIR Mhealth Uhealth. 2019 Sep 3;7(9):e14474. doi: 10.2196/14474 (PMC6751099; doi:10.2196/14474)
Supplement: Multimedia Appendix 4 [file mhealth_v7i9e14474_app4.pdf]

Multimedia Appendix 4

Appendix with the results of Dunn-Bonferroni post-hoc tests for the examination of the differences between the devices:

Attractive design ratings for each device over the female subjects (N=11)

Pairwise Comparisons

Sample 1-Sample 2	Test Statistic	Std. Error	Std. Test Statistic	Sig.	Adj. Sig. (P)	Effect size (r)
g.LADYbird-g.SAHARA	.273	.921	.296	.77	1.00	0.03
g.LADYbird-Trilobite	.591	.921	.642	.52	1.00	0.07
g.LADYbird-BR8+	2.227	.921	2.418	.02	.33	0.28
g.LADYbird-MindCap	2.545	.921	2.763	.006	.12	0.31
g.LADYbird-Jellyfish	2.955	.921	3.208	.001	.03	0.37
g.LADYbird-EPOC	3.818	.921	4.145	<.001	.001	0.47
g.SAHARA-Trilobite	-.318	.921	-.345	.73	1.00	0.04
g.SAHARA-BR8+	1.955	.921	2.122	.03	.71	0.24
g.SAHARA-MindCap	2.273	.921	2.467	.01	.29	0.28
g.SAHARA-Jellyfish	2.682	.921	2.911	.004	.08	0.33
g.SAHARA-EPOC	3.545	.921	3.849	<.001	.002	0.44
Trilobite-BR8+	1.636	.921	1.776	.08	1.00	0.20
Trilobite-MindCap	1.955	.921	2.122	.03	.71	0.24
Trilobite-Jellyfish	2.364	.921	2.566	.01	.22	0.29
Trilobite-EPOC	3.227	.921	3.504	<.001	.01	0.40
BR8+-MindCap	.318	.921	.345	.73	1.00	0.04
BR8+-Jellyfish	.727	.921	.790	.43	1.00	0.09
BR8+-EPOC	-1.591	.921	-1.727	.08	1.00	0.20
MindCap-Jellyfish	-.409	.921	-.444	.66	1.00	0.05
MindCap-EPOC	-1.273	.921	-1.382	.17	1.00	0.16
Jellyfish-EPOC	-.864	.921	-.938	.35	1.00	0.11

Each row tests the null hypothesis that the Sample 1 and Sample 2 distributions are the same.

Asymptotic significances (2-sided tests) are displayed. The significance level is .05.
