# Supplementary material for: User Experience of 7 Mobile Electroencephalography Devices: Comparative Study
Source: JMIR Mhealth Uhealth. 2019 Sep 3;7(9):e14474. doi: 10.2196/14474 (PMC6751099; doi:10.2196/14474)
Supplement: Multimedia Appendix 5 [file mhealth_v7i9e14474_app5.pdf]

Multimedia Appendix 5

Appendix with the results of Dunn-Bonferroni post-hoc tests for the examination of the differences between the devices:

Practicability ratings for each device over all subjects (N=24)

Pairwise Comparisons

Sample 1-Sample 2	Test Statistic	Std. Error	Std. Test Statistic	Sig.	Adj. Sig. (P)	Effect size (r)
g.LADYbird-Trilobite	2.104	.624	3.374	.001	.02	0.26
g.LADYbird-g.SAHARA	2.708	.624	4.343	<.001	<.001	0.34
g.LADYbird-BR8+	3.167	.624	5.078	<.001	<.001	0.39
g.LADYbird-EPOC	3.688	.624	5.913	<.001	<.001	0.46
g.LADYbird-MindCap	3.833	.624	6.147	<.001	<.001	0.47
g.LADYbird-Jellyfish	3.896	.624	6.247	<.001	<.001	0.48
Trilobite-g.SAHARA	.604	.624	.969	.33	1.00	0.07
Trilobite-BR8+	1.063	.624	1.704	.09	1.00	0.13
Trilobite-EPOC	1.583	.624	2.539	.01	.23	0.20
Trilobite-MindCap	1.729	.624	2.773	.006	.12	0.21
Trilobite-Jellyfish	1.792	.624	2.873	.004	.09	0.22
g.SAHARA-BR8+	.458	.624	.735	.46	1.00	0.06
g.SAHARA-EPOC	.979	.624	1.570	.12	1.00	0.12
g.SAHARA-MindCap	1.125	.624	1.804	.07	1.00	0.14
g.SAHARA-Jellyfish	1.188	.624	1.904	.06	1.00	0.15
BR8+-EPOC	-.521	.624	-.835	.40	1.00	0.06
BR8+-MindCap	.667	.624	1.069	.29	1.00	0.08
BR8+-Jellyfish	.729	.624	1.169	.24	1.00	0.09
EPOC-MindCap	.146	.624	.234	.82	1.00	0.02
EPOC-Jellyfish	.208	.624	.334	.74	1.00	0.03
MindCap-Jellyfish	-.063	.624	-.100	.92	1.00	0.01

Each row tests the null hypothesis that the Sample 1 and Sample 2 distributions are the same.

Asymptotic significances (2-sided tests) are displayed. The significance level is .05.
