# Supplementary material for: User Experience of 7 Mobile Electroencephalography Devices: Comparative Study
Source: JMIR Mhealth Uhealth. 2019 Sep 3;7(9):e14474. doi: 10.2196/14474 (PMC6751099; doi:10.2196/14474)
Supplement: Multimedia Appendix 6 [file mhealth_v7i9e14474_app6.pdf]

Multimedia Appendix 6

Appendix with the results of Dunn-Bonferroni post-hoc tests for the examination of the differences between the devices:

Practicability ratings for each device over subjects with negative attitude towards technology (N=12)

Pairwise Comparisons

Sample 1-Sample 2	Test Statistic	Std. Error	Std. Test Statistic	Sig.	Adj. Sig. (P)	Effect size (r)
g.LADYbird-Trilobite	2.875	.882	3.260	.001	.02	0.36
g.LADYbird-g.SAHARA	3.042	.882	3.449	.001	.01	0.38
g.LADYbird-EPOC	3.417	.882	3.874	<.001	.002	0.42
g.LADYbird-BR8+	3.458	.882	3.921	<.001	.002	0.43
g.LADYbird-MindCap	3.958	.882	4.488	<.001	<.001	0.49
g.LADYbird-Jellyfish	3.958	.882	4.488	<.001	<.001	0.49
Trilobite-g.SAHARA	.167	.882	.189	.85	1.00	0.02
Trilobite-EPOC	.542	.882	.614	.54	1.00	0.07
Trilobite-BR8+	.583	.882	.661	.51	1.00	0.07
Trilobite-MindCap	1.083	.882	1.228	.22	1.00	0.13
Trilobite-Jellyfish	1.083	.882	1.228	.22	1.00	0.13
g.SAHARA-EPOC	.375	.882	.425	.67	1.00	0.05
g.SAHARA-BR8+	.417	.882	.472	.64	1.00	0.05
g.SAHARA-MindCap	.917	.882	1.039	.30	1.00	0.11
g.SAHARA-Jellyfish	.917	.882	1.039	.30	1.00	0.11
EPOC-BR8+	.042	.882	.047	.96	1.00	0.01
EPOC-MindCap	.542	.882	.614	.54	1.00	0.07
EPOC-Jellyfish	.542	.882	.614	.54	1.00	0.07
BR8+-MindCap	.500	.882	.567	.57	1.00	0.06
BR8+-Jellyfish	.500	.882	.567	.57	1.00	0.06
MindCap-Jellyfish	.000	.882	.000	1.00	1.00	0.00

Each row tests the null hypothesis that the Sample 1 and Sample 2 distributions are the same.
Asymptotic significances (2-sided tests) are displayed. The significance level is .05.
