# Supplementary material for: User Experience of 7 Mobile Electroencephalography Devices: Comparative Study
Source: JMIR Mhealth Uhealth. 2019 Sep 3;7(9):e14474. doi: 10.2196/14474 (PMC6751099; doi:10.2196/14474)
Supplement: Multimedia Appendix 7 [file mhealth_v7i9e14474_app7.pdf]

Multimedia Appendix 7

Appendix with the results of Dunn-Bonferroni post-hoc tests for the examination of the differences between the devices:

Practicability ratings for each device over subjects with positive attitude towards technology (N=12)

Pairwise Comparisons

Sample 1-Sample 2	Test Statistic	Std. Error	Std. Test Statistic	Sig.	Adj. Sig. (P)	Effect size (r)
g.LADYbird-Trilobite	1.333	.882	1.512	.13	1.00	0.16
g.LADYbird-g.SAHARA	2.375	.882	2.693	.007	.15	0.29
g.LADYbird-BR8+	2.875	.882	3.260	.001	.02	0.36
g.LADYbird-MindCap	3.708	.882	4.205	<.001	.001	0.46
g.LADYbird-Jellyfish	3.833	.882	4.347	<.001	<.001	0.47
g.LADYbird-EPOC	3.958	.882	4.488	<.001	<.001	0.49
Trilobite-g.SAHARA	1.042	.882	1.181	.29	1.00	0.13
Trilobite-BR8+	1.542	.882	1.748	.08	1.00	0.19
Trilobite-MindCap	2.375	.882	2.693	.007	.15	0.29
Trilobite-Jellyfish	2.500	.882	2.835	.005	.10	0.31
Trilobite-EPOC	2.625	.882	2.976	.003	.06	0.32
g.SAHARA-BR8+	.500	.882	.567	.57	1.00	0.06
g.SAHARA-MindCap	1.333	.882	1.512	.13	1.00	0.16
g.SAHARA-Jellyfish	1.458	.882	1.654	.10	1.00	0.18
g.SAHARA-EPOC	1.583	.882	1.795	.07	1.00	0.20
BR8+-MindCap	.833	.882	.945	.35	1.00	0.10
BR8+-Jellyfish	.958	.882	1.087	.28	1.00	0.12
BR8+-EPOC	-1.083	.882	-1.228	.22	1.00	0.13
MindCap-Jellyfish	-.125	.882	-.142	.89	1.00	0.02
MindCap-EPOC	-.250	.882	-.283	.78	1.00	0.03
Jellyfish-EPOC	-.125	.882	-.142	.89	1.00	0.02

Each row tests the null hypothesis that the Sample 1 and Sample 2 distributions are the same.

Asymptotic significances (2-sided tests) are displayed. The significance level is .05.
